# Hybrid Polymer/Metal Oxide Thin Films for High Performance, Flexible Transistors

**DOI:** 10.3390/mi11030264

**Published:** 2020-03-04

**Authors:** Jae Won Jeong, Hye Suk Hwang, Dalsu Choi, Byung Chol Ma, Jaehan Jung, Mincheol Chang

**Affiliations:** 1 Department of Polymer Engineering, Graduate School, Chonnam National University, Gwangju 61186, Korea; sjndow1221@naver.com; 2 Alan G. MacDiarmid Energy Research Institute, Chonnam National University, Gwangju 61186, Korea; hshwang33@gmail.com; 3 Department of Chemical Engineering, Myongji University, Yongin-si, Gyeonggido 17058, Korea; dalsuchoi@mju.ac.kr; 4 School of Chemical Engineering, Chonnam National University, Gwangju 61186, Korea; 5 Department of Materials Science and Engineering, Hongik University, Sejong 30016, Korea; 6 School of Polymer Science and Engineering, Chonnam National University, Gwangju 61186, Korea

**Keywords:** flexible transistors, polymers, metal oxides, nanocomposites, dielectrics, active layers

## Abstract

Metal oxides (MOs) have garnered significant attention in a variety of research fields, particularly in flexible electronics such as wearable devices, due to their superior electronic properties. Meanwhile, polymers exhibit excellent mechanical properties such as flexibility and durability, besides enabling economic solution-based fabrication. Therefore, MO/polymer nanocomposites are excellent electronic materials for use in flexible electronics owing to the confluence of the merits of their components. In this article, we review recent developments in the synthesis and fabrication techniques for MO/polymer nanocomposite-based flexible transistors. In particular, representative MO/polymer nanocomposites for flexible and transparent channel layers and gate dielectrics are introduced and their electronic properties—such as mobilities and dielectric constant—are presented. Finally, we highlight the advances in interface engineering and its influence on device electronics.

## 1. Introduction

Thin-film transistors (TFTs) are the crucial elements in flat-panel display (FPD) applications, including both active matrix liquid crystal displays (AMLCDs) and active matrix organic light emitting diode (AMOLEDs) displays [1,2,3]. In recent years, the traditional amorphous Si (a-Si) TFT technology has achieved higher resolutions, larger screen sizes, and lower power consumptions in FPDs [4,5]. However, the demand for transparent, flexible, and stretchable optoelectronic devices remains, which requires further advancement in crucial component materials, including the semiconductor, the dielectric, and the conductor, as well as the substrates [6,7,8,9,10,11,12].

As mechanically flexible and durable semiconductors as well as gate dielectrics, metal oxides (MOs) such as In_2_O_3_, ZrO_2_, Al_2_O_3_, and TiO_2_ are now expected to be one of the most promising materials for next generation display technologies, because of their high carrier mobility, good transparency, excellent uniformity, and reasonable electrical reliability/stability [13,14,15,16,17,18,19,20]. More importantly, MOs exhibit high carrier mobilities even in the amorphous state and satisfactory environmental stability [21,22]. It is worth noting that the amorphous phase is favorable for use in flexible devices compared to the crystalline phase, as crystalline materials tend to crack when folded. Indium oxide (In_2_O_3_) is the most heavily investigated metal oxide both as a conductor and a semiconductor, since the extensive 5s orbital overlap leads to a broad conduction band with high electron mobility even in the amorphous state [23]. Furthermore, their large bandgap ensures optical transparency. The conventional strategy to achieve optimal conductivity in In_2_O_3_ is to chemically dope the compound with various cations such as Sn, Ga, La, or Sc [24,25,26]. For example, ITO (indium−tin−oxide) exhibits excellent transparency with high conductivity since the Sn ion enhances the carrier density by donating free electrons to the lattice due to the difference in oxidation state between In^3+^ and Sn^4+^ [27,28]. In IGZO (indium−gallium−zinc−oxide), Ga forms stronger chemical bonds with oxygen and suppresses the formation of oxygen deficiencies and free electrons, thereby serving the role of a “stabilizer” or a “suppressor” [29]. Currently, commercially available metal oxide (semi)conductor films are primarily fabricated via capital-intensive vacuum vapor deposition processes, such as sputtering or thermal evaporation, thereby limiting the large scale and economic production of MO films. Post-annealing processes to enhance charge carrier mobility require high processing temperatures to induce metal–oxygen–metal lattice formation. However, such high temperatures are not suitable for fabrication of MO on soft polymeric substrates such as polyimide (PI), polyethylene naphthalate, polyethylene terephthalate, polydimethylsiloxane, and parylene [29,30,31]. Moreover, mechanical toughness is also required for the use of inflexible and foldable devices.

In this context, novel processing techniques for fabricating flexible MO films with high charge carrier mobilities is in great demand. Organic polymers such as poly(4-vinylphenol) (PVP), polytetrafluoroethylene (PTFE), and polyethylenimine (PEI), therefore, have been utilized as flexible matrices with various MO fillers due to their merits such as flexibility, light-weight, durability, and solution-processability [32,33,34,35]. Using MO/polymer nanocomposites, the films can be easily fabricated via solution-based fabrication processes including spin-casting and roll-to-roll. 

This review seeks to summarize the recent progress in the synthesis and fabrication techniques of MO/polymer nanocomposites for flexible transistors. In particular, the synthesis of metal oxides/polymers nanocomposites for flexible channel layers and gate dielectrics, alongside their electronic properties such as mobilities and dielectric constant, are presented. Furthermore, advances in interface engineering and their influence on device electronics are highlighted.

## 2. Synthesis of Metal Oxides

**Figure 1:**
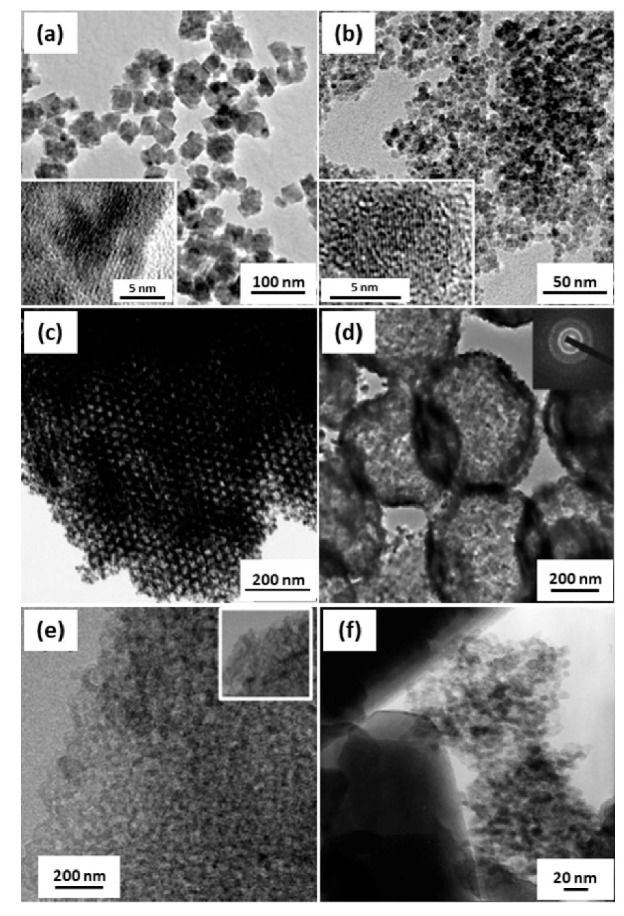
(**a**–**f**) A set of TEM images of diverse MO nanostructures: (**a**) MnO and (**b**) Fe_3_O_4_ nanoparticles fabricated via microwave-assisted synthesis (Reproduced from [42], Copyright 2008 Royal Society of Chemistry C), (**c**) porous SnO_2_ aerosols prepared via sol–gel method (reproduced with permission from [41], Copyright 2005 Wiley-VCH Verlag GmbH & Co. KGaA, Weinheim), (**d**) ZnO hollow spheres synthesized via hydrothermal synthesis (reproduced with permission from [40], Copyright 2008 American Chemical Society), (**e**) In_2_O_3_ nanoparticles prepared via anodization-precipitation (reproduced with permission from [37], Copyright 2018 American Chemical Society), and (**f**) TiO_2_ nanoparticle layer on SiO_2_ prepared via CVD, respectively (reproduced with permission from [43], Copyright 2001 Elsevier).

New techniques have continued to emerge for the synthesis of MO nanostructures with controlled shape, size, and composition, because these factors play an important role in any application [36,37]. In particular, the morphology of MOs are strongly dependent on the synthetic route [38,39]. Therefore, it is critical to select an appropriate synthetic technique to achieve the desired morphology of MOs. In general, a lot of approaches have been reported for the synthesis of various MO nanostructures (Figure 1), including precipitation, hydrothermal, sol–gel methods, microwave-assisted synthesis, and chemical vapor deposition (CVD) [37,40,41,42,43].

The earliest technique that was developed to synthesize inorganics is the precipitation method. The primary merit of this strategy is its ease of scalability in the synthesis of MOs for commercialization [44]. In a typical process, the precipitation of sparingly soluble hydroxides takes place from an aqueous solution on the addition of a precipitating agent (anion) or ligand (e.g., urea, hexamethylenetetramine, and KOH) to the metal salt solution containing a cation. Subsequently, the precipitated hydroxides are decomposed to metal oxides [45]. It is very difficult to control the uniformity of the product structures via the precipitation approach, owing to a lack of understanding of major processing steps, namely nucleation and growth [46].

Hydrothermal methods are very simple and capable of generating MOs with diverse morphologies, such as spheres, rods, wires, and cones [47,48,49,50]. During synthesis, a heterogeneous reaction occurs in an aqueous solvent containing NaOH, KOH, HCl, HNO_3_, H_2_SO_4_, etc. under a particular pressure and temperature [51]. The major benefits of hydrothermal syntheses are its low processing temperature, reduced aggregation of the products, homogeneous crystallinity of the products, and satisfactory uniformity in composition and purity of the products [52,53]. Occasionally, surfactants such as cetyltrimethylammonium bromide, sodium dodecyl sulfate, and polyvinylpyrrolidine (PVP) are utilized—the surfactant molecules selectively adhere onto the polar surface of the MO crystals, controlling the shape and growth behavior of MO particles [54,55,56].

**Figure 2:**
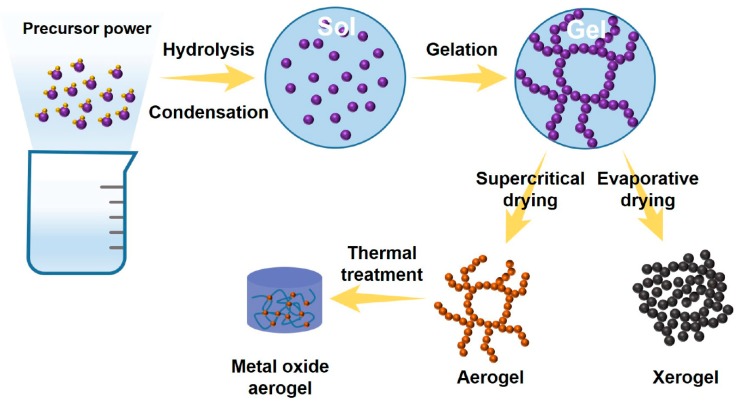
Reaction routes for the production of MO nanostructures by the sol–gel method.

Sol–gel is a general, versatile, and powerful approach for the synthesis of single- or multiple-component MO nanostructures in the form of thin films, powders, and porous materials. This approach is a cost effective and low-temperature process that enables the production of MO nanostructures with high homogeneity and compositional purity [57,58]. Metal alkoxides [M(OR)_3_] are primarily used as a precursor to prepare MOs due to their propensity to form homogeneous solution in a variety of solvents in the presence of other alkoxides or metallic derivatives and also due to their reactivity toward nucleophiles such as water [59]. The sol–gel process involves several important steps, such as hydrolysis and condensation, gelation, and drying (Figure 2). Typically, metal precursors such as metal alkoxides and metal chlorides undergo the reactions of hydrolysis and partial condensation to form a colloidal solution. Subsequently, three-dimensional gels are formed immediately via polycondensation of the hydrolyzed precursors. Finally, the resultant gels are converted to xerogel or aerogel based on the method of drying (i.e., supercritical drying or ambient drying) and, furthermore, to the desired MO materials via a thermal treatment. The sol–gel technique can be divided into two routes—namely the aqueous sol–gel and the nonaqueous sol–gel methods. The aqueous sol–gel method requires oxygen for the formation of MOs, which is generally provided by the water solvent. However, this approach is not suitable for the production of MO nanomaterials because the crucial steps (i.e., hydrolysis, condensation, and drying) take place simultaneously and thus result in the formation of bulk MOs [39,60]. In contrast, solvents such as alcohols, ketones, and aldehydes are used to provide the oxygen necessary for the formation of MOs via the nonaqueous sol–gel method [39,61,62,63]. Additionally, this approach is suitable for the production of MO nanomaterials, rather than their bulk counterparts. The organic solvents serve as important components by controlling morphology, particle size, surface properties, and composition of the resultant MO materials [64].

Microwave-assisted synthesis is an approach that applies microwave radiation to chemical reactions for the production of MO nanostructures. This method could allow more efficient, rapid, and homogenous heating of reaction mixtures, thereby accelerating the synthesis of MO nanostructures [65]. Furthermore, the formation of fine MO nanocrystals is enabled by the use of microwave radiation due to the highly focused local heating that can be achieved [66]. Moreover, the microwave-assisted approach can produce a wide range of MO nanostructures, including nanoflakes [67], nanosheets [68], and nanoflowers [69]. However, the microwave-assisted synthesis possesses some drawbacks, such as the high cost of microwave reactor and the limited penetration depth of microwave radiation, indicative of restricted scalability for the commercial synthesis of MO nanoparticles [70].

## 3. Metal Oxide/Polymer Hybrid Films in Transistors 

### 3.1. Active Channel Layers

Although metal oxides (MOs) exhibit high carrier mobilities and good environmental stability even in the amorphous state, their application in flexible and stretchable devices has been rather limited [25,71,72,73]. This is because polycrystalline materials suffer from crack formation at the grain boundaries leading to drastic deterioration of structural integrity [74,75,76,77,78]. Recently, amorphous metal oxides (MOs) have been prepared to improve flexibility. However, they are still vulnerable to mechanical stress, yielding cracks under repeated mechanical deformation. On the other hand, polymers exhibit flexibility, solution-processability, and excellent compatibility with organic substrates or active layers [79,80]. In this context, organic–inorganic nanocomposites can gain the synergetic advantages of these two components—namely, mechanical toughness, flexibility, and high mobility [81,82]. Moreover, the incorporation of polymers with MOs successfully inhibits the formation of the crystalline phase which is detrimental to flexible substrates. It should be noted that in general, the trade-off between the mechanical properties and electrical properties is observed in MO/polymer nanocomposites. In other words, while incorporation of polymers to MO films gives rise to an increase in flexibility, it leads to a potential reduction in electrical properties due to phase separations and lack of interconnectivity of MO domains in the resultant composite films. To overcome the issue, various strategies that can improve the interconnectivity of MO domains within the composite films have emerged in recent years, including engineering of weight fraction, surface modification, and morphology control of MO nanoparticles [83,84,85,86].

**Figure 3:**
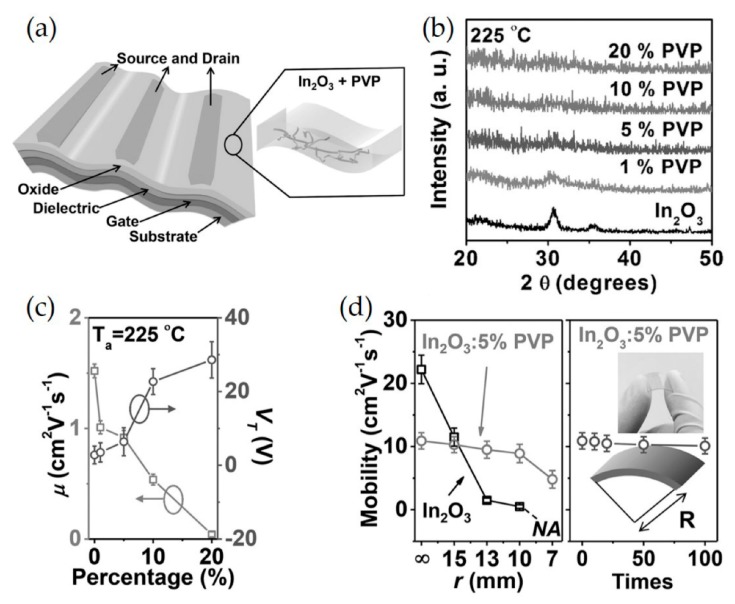
(**a**) Schematic representation of the flexible, transparent TFT structure based on a metal oxide:polymer (In_2_O_3_: *x*% PVP) semiconductor blend. (**b**) X-ray diffraction (XRD) patterns of In_2_O_3_: polymer films with various PVP concentrations: annealing at 225 °C. (**c**) TFT mobility and threshold voltage for In_2_O_3_: polymer films having different PVP concentrations, processed at 225 °C. (**d**) Dependence of TFT mobilities on bending radius of both neat In_2_O_3_ TFTs and all-amorphous In_2_O_3_: 5% PVP TFTs (left), and mobilities on all-amorphous TFT bending cycles at a radius of 10 mm. Inset: Optical image of transparent flexible TFTs. Reproduced with permission from [91], Copyright 2015 Wiley-VCH Verlag GmbH & Co. KGaA, Weinheim.

To improve mechanical flexibility of metal oxide (MO) films, polymers such as poly(4-vinylphenol) (PVP), polytetrafluoroethylene (PTFE), and polyethylenimine (PEI) were utilized as doping agents to improve flexibility, as well as to form the amorphous phase of MO [35,87,88,89,90]. For example, Yu et al. developed a new low temperature approach to high-mobility amorphous metal oxide semiconductor films via doping with an insulating polymer, poly(4-vinylphenol) (PVP), to fabricate amorphous MO: polymer blend composites, as depicted in Figure 3a [91]. It should be noted that PVP possesses excellent solubility in the In_2_O_3_ precursor solution and their hydroxyl groups favor coordination with the MO lattice. Such an approach effectively prevents crystallization, controls the carrier concentration in the In_2_O_3_ channel, and retains conducting pathways for efficient charge transportation. In greater detail, all-amorphous and transparent bottom-gate top-contact thin-film transistors (TFTs) were fabricated via spin-coating of In_2_O/PVP precursor solutions on AryLite substrates and annealing at 225–250 °C. They exhibited high transparency (< 80%) and low sheet resistance (< F Ω sq^−1^). In_2_O_3_: 5% PVP TFTs exhibited electron mobilities of 11 cm^2^ V^−1^ s^−1^. As the amount of PVP content increases, the In_2_O_3_ films become amorphous even with 1 wt % of PVP, as confirmed by grazing incidence X-ray diffraction (GIXRD)(Figure 3b). V_T_ shifts to a positive value and the mobility slightly decreases upon incorporation of PVP, as evidenced in Figure 3c, owing to the carrier concentration modulation from PVP-induced electron traps. The bending/relaxation measurement is to characterize durability of flexible films. A film is bended and relaxed several times with defined radius and then electronic properties are measured. It is worth noting that smaller radius is for more harsh condition; the bending radii that are required for flexible, rollable, and foldable displays are 0r, 10r, and 1r, respectively [92]. The bending tests indicate that the In_2_O_3_: 5%PVP hybrid films exhibit only a slight decrease in the mobility from 10.9 to 8.9 cm^2^ V^−1^ s^−1^ as the bending radius decreases to 10 mm, while the pristine In_2_O_3_ films, in stark contrast, show dramatic deterioration of the mobility from 22.2 to 0.5 cm^2^ V^−1^ s^−1^ (Figure 3d). Importantly, the value retains up to ca. 90% of their performance even after undergoing repeated mechanical stress (bending/relaxing 100 times). 

**Figure 4:**
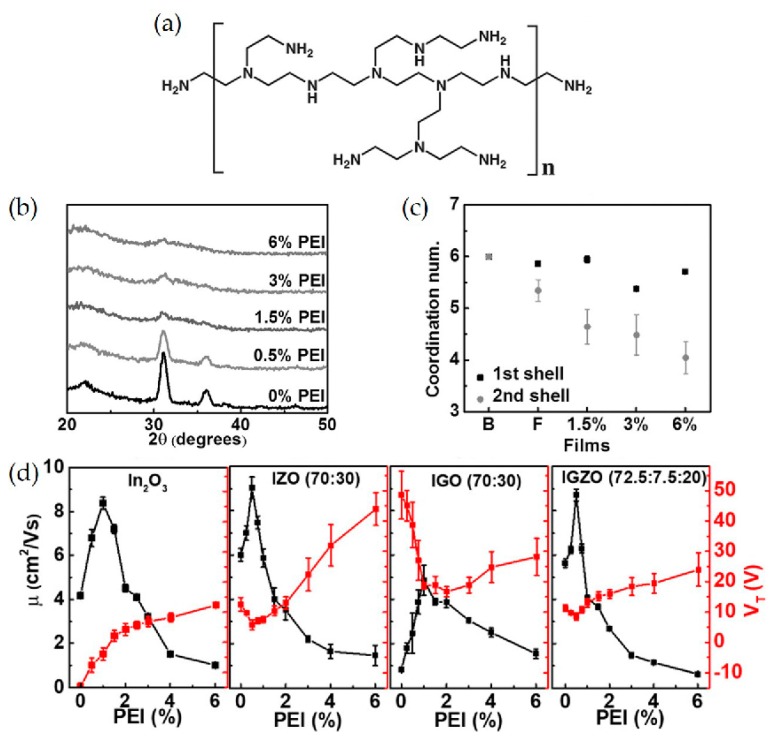
(**a**) Chemical structure of PEI. (**b**) GIXRD patterns of In_2_O_3_: x% PEI blend films with differing PEI concentrations. (**c**) Derived coordination number, In-O bond lengths for the indicated films. (**d**) TFT mobility and threshold voltage for In_2_O_3_: *x* wt % PEI (250 °C), IZO: *x* wt % PEI, IGO: *x* wt % PEI, and IGZO: *x* wt % PEI, as a function of the polymer concentration. T_annealing_ = 300 °C. Reproduced with permission from [93], Copyright, 2016 Wiley-VCH Verlag GmbH & Co. KGaA, Weinheim.

Polyethylenimine (PEI) is a commercially available polymer capable of efficient n-doping due to the electron-donating nature of the tertiary amine groups (Figure 4a). PEI electron doping has been reported for several organic semiconductors and is widely used in organic photovoltaic cells and transistors to enhance the charge transportation in other organic materials. In this context, Huang et al. fabricated In_2_O_3_ / PEI TFT devices via doping of metal oxides with PEI [93]. Doping of In_2_O_3_ with PEI effectively prevents crystallization of MOs, controls the carrier concentration in the In_2_O_3_ channel, and increases the electron mobility of the In_2_O_3_ matrix. In greater detail, a PEI-doped In_2_O_3_ blend (i.e., aqueous PEI-In_2_O_3_ precursor solutions) was coated on Si substrates with 300 nm SiO_2_, followed by annealing at 250 °C for 30 min. The addition of PEI successfully inhibits the formation of crystalline structure, which is unfavorable for application in flexible devices as characterized by GIXRD in Figure 4b. The characteristic peaks at 22.1°, 31.1°, 36.0°, and 46.3° ascribed to crystalline In_2_O_3_ are strongly suppressed even with a PEI concentration of >1%. Extended X-ray absorption fine structure (EXAFS) measurements correlate the effect of PEI with the TFT mobility. The coordination number (CN) of In-O at the first shell remains intact independent of the PEI doping concentrations, while the second shell CN exhibits the PEI content dependency, decreasing from 6 to 4.05 as the PEI concentration increases (Figure 4c). This indicates that PEI disrupts the formation of lattices, and thus electron conduction pathways. The devices fabricated with polymer concentration of 1–1.5% resulted in excellent mobility up to 9 cm^2^ V^−1^ s^−1^ and high on/off ratio of 10^7^, while that fabricated with pure In_2_O_3_ only exhibited a maximum value of 9 cm^2^ V^−1^ s^−1^ (Figure 4d). It is because the electron donating nature of PEI results in doping of In_2_O_3_ and matrix film microstructure tuning, yielding high mobilities alongside optimal off-current (*I*_off_) and threshold voltage (*V*_th_). 

The same research group also investigated the charge transportation and film microstructure evolution of PEI-doped amorphous Zn- and/or Ga-incorporated In_2_O_3_ thin films [94]. PEI doping generality was expanded from binary In_2_O_3_ to ternary (e.g., In + Zn in IZO, In + Ga in IGO) and quaternary (e.g., In + Zn + Ga in IGZO) metal oxide matrices. PEI-metal oxide precursor solutions on Si wafers with 300 nm wide thermally grown SiO_2_ layers were first spin-casted and then thermally annealed at 300 °C. In this study, the effect of PEI doping concentration and the addition of secondary ions (Ga and Zn) to In_2_O_3_ on the device performance was investigated. It was found that the incorporation of Zn and PEI in In_2_O_3_ and IGO led to an increase in the surface roughness, thereby degrading the charge transport properties. The crystallinity of In_2_O_3_ or IG(Z)O was effectively suppressed and it was observed to monotonically decrease as the PEI concentration was increased. The layer formed adjacent to the dielectric improves the efficiency of charge transportation in a channel when PEI content is low because of trap prefilling. When PEI concentration exceeds a certain threshold, the mobility of the resulting devices begins to decrease due to the disruptions in film continuity and increased trap sites.

**Figure 5:**
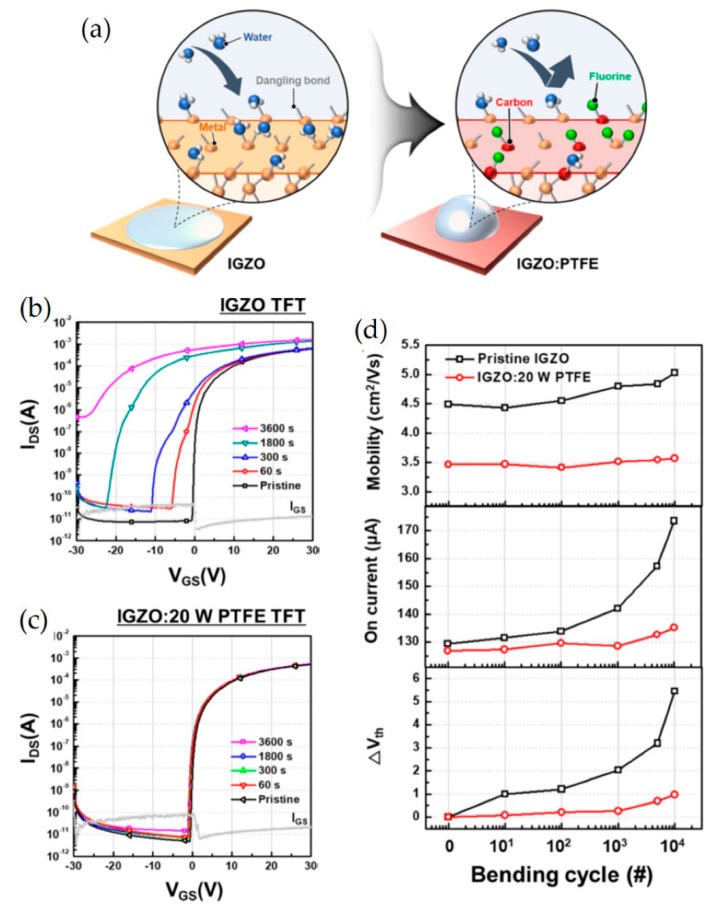
(**a**) Schematic illustration of improved hydrophobicity of the IGZO: PTFE film. Transfer characteristics of (**b**) the IGZO TFT and (**c**) the IGZO: 20 W PTFE TFT upon exposure to water for different times. (**d**) Variations of mobility (μ_FE_), on-current, and *V*_th_ for IGZO and IGZO: 20 W PTFE TFTs with respect to bending cycles. Reproduced permission from [95], Copyright, 2018, American Chemical Society.

Na et al. demonstrated flexible IGZO:PTFE TFTs with improved stability and endurability against water exposure using the facile method of blending the MO semiconductor with polytetrafluoroethylene (PTFE) via plasma polymerization [95]. In greater detail, the IGZO: PTFE layer was co-sputtered with radio frequency magnetron sputtering processes. The hydrophobic nature of PTFE enhances device performance (μ_FE_ exceeding 10 cm^2^ V^−1^ s^−1^) and stability (a *V*_th_ shift of 0.68 V after an hour of immersion in water) by preventing the adsorption of water molecules on the back surface of TFTs (Figure 5a). Such an approach also improves the electrical stability of IGZO: PTFE TFTs in positive bias stress (PBS), positive bias temperature stress (PBTS), positive bias illumination stress (PBIS), negative bias stress (NBS), negative bias temperature stress (NBTS), and negative bias illumination stress (NBIS) stability tests. Indeed, *V*_th_ of IGZO: PTFE TFT remains steady, with only a shift of 0.68 V, while that of IGZO TFTs exhibits significant negative shifts by 12.17 V, as depicted in Figure 5b,c. The improved mechanical flexibility resulting from the soft nature of PTFE enhances the mechanical durability, as depicted in Figure 5d. Specifically, the IGZO: PTFE TFT can retain its performance with no substantial change in its electrical characteristics (a *V*_th_ shift of 0.89 V from 3.95 to 3.06 V) over 10000 bending cycles with a bending radius of 5 mm. In contrast, the IGZO TFTs exhibit a significant V_th_ shift of 5.45 V, from 3.07 to −2.38 V. 

**Figure 6:**
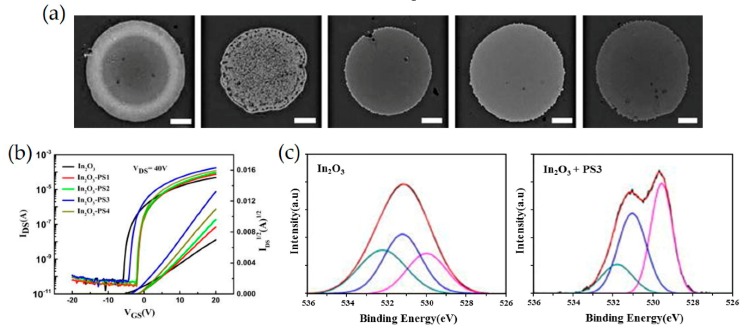
(**a**) Optical microscopy images of In_2_O_3_ deposition (right to left) without PS, with PS1, with PS2, with PS3, and with PS4. The scale bar is 100 μm. (**b**) Transfer characteristics of inkjet-printed TFTs. (**c**) X-ray photoelectron spectroscopy (XPS) of O 1s spectra. Reproduced with permission from [96], Copyright 2018, American Institute of Physics.

Sun et al. reported a strategy to control the geometry and enhance device performance of inkjet-printed MOTFT arrays via the addition of an insulating polymer to the precursor solution prior to film deposition [96]. To prevent the formation of a non-uniform geometry during inkjet-printing, the polymer additive, polystyrene (PS) was utilized. It was reported that the addition of high viscous polymers is favorable to eliminate coffee ring effects by significantly reducing the solute mobility and thus suppressing outward capillary flow of solute to the edge. In detail, PS, with different molecular weights ranging from 2000 to 2,000,000, was mixed with indium precursors (i.e., indium nitrate hydrate) and then printed on silicon substrates, followed by annealing at 225 °C for 1 h. Interestingly, 202 with an increase in PS MW, the coffee ring effect gradually faded, as measured by optical microscope in Figure 6a. The change in surface morphology by varying PS MW is attributed to the suppressed capillary flow and the Marangoni effect. The relative viscosities of In_2_O_3_/PS precursor solutions to those of pristine In_2_O_3_ solution are 1.02, 1.17, 1.39, and 1.31 for PS with MW of 2000, 20,000, 200,000, and 2,000,000, respectively. Evidently, the use of PS with MW of 20,000 results in smooth films as the increased viscosity inhibits the capillary flow, thus facilitating the depinning of the contact line. The incorporation of PS results in the improvement of carrier mobility from 4.2 cm^2^ V^−1^ s^−1^ up to 13.7 cm^2^ V^−1^ s^−1^ as PS MW increases from 2000 to 2,000,000, which is about three times that of the pristine In_2_O_3_ TFTs (Figure 6b). The trap densities for pristine, PS Mw of 2000, 20,000, 200,000, and 2,000,000 were 2.4 × 10^12^, 1.2 × 10^12^, 1.1 × 10^12^, 1.0 × 10^12^, and 1.1 × 10^12^ cm^−2^ eV^−1^, respectively. XPS characterization shows that the incorporation of PS obviously impacts local bonding of MO:PS blends, thereby increasing M-O concentration (Figure 6c). Grazing incidence X-ray diffraction indicates that the addition of PS favors the formation of amorphous phase and enhances the M-O lattice contents, both of which facilitate the carrier transportation. 

### 3.2. Dielectric Layers

MOs have been considered to be a crucial component in thin-film electronic systems due to their outstanding electrical and mechanical properties [97,98,99,100,101,102,103,104]. However, the MOs lack flexibility, which limits their use in flexible electronics [105]. Thus far, most thin high-k inorganic metal oxide dielectrics have been fabricated via conventional vacuum-based techniques including pulsed laser deposition (PLD), atomic layer deposition (ALD), magnetron sputtering, and e-beam evaporation. However, these methods are costly and unsuitable to produce large-area flexible oxide electronics. For example, high-quality gate dielectric SiO_2_ films are produced via expensive vacuum-based plasma-enhanced chemical vapor deposition (PECVD) at high temperatures above 300 °C. It is worth noting that such high temperatures may cause deformation or warping of flexible substrates. Although high performances have been achieved, flexibility and stability still limit their application in real products such as wearable devices. Moreover, dielectric layers (e.g., SiO_2_) are vulnerable to mechanical stress notwithstanding their extremely thin width, yielding cracks or delamination under mechanical deformation even at small bending radii. In this context, organic dielectrics have garnered substantial attention in the area of flexible devices owing to their flexibility, mechanical stability, low temperature, easy solution processability, and excellent compatibility with flexible organic substrates, despite their low k value [105,106,107,108,109,110]. Therefore, hybridization of organic and inorganic materials can lead to the improvement in flexibility, dielectric constant, and mechanical toughness of gate dielectric materials [111,112,113,114,115,116,117,118,119]. The transistor parameters critically depend on the interface formed between dielectric and semiconductor layers since the trapped charges strongly impact the electrical behavior. The hybrid gate dielectrics tend to be compatible with either organic or inorganic semiconductors. As the inorganic constituent, high-k inorganic nanoparticles such as ZrO_2_, Al_2_O_3_, Y_2_O_3_, Ta_2_O_5_, and TiO_2_ were usually embedded in a polymeric matrix such as poly(methylmethacrylate) (PMMA) and poly(vinylpyrrolidone) [120,121,122]. However, the inorganic nanoparticles tend to agglomerate, increasing the surface roughness of the hybrid layers, resulting in high gate leakage current and low on/off current ratio. In this section, the development of hybrid organic–inorganic composites with low power consumption, low operating voltage, and compatibility with transparent flexible electronics for the use in dielectric layers will be summarized.

**Figure 7:**
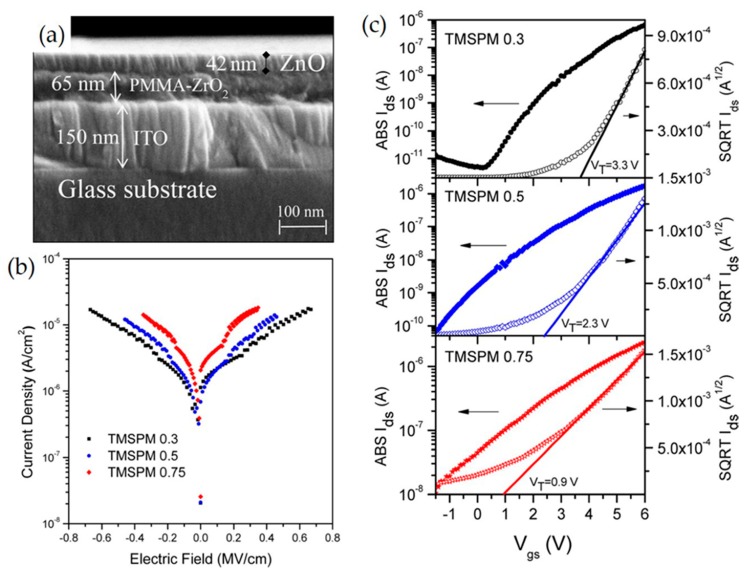
(**a**) SEM image of the TFT cross section, where the PMMA−ZrO_2_ layer was deposited with 1:0.3:1 molar ratio. (**b**) Leakage current density vs. electric field of the PMMA–ZrO_2_ hybrid layers deposited with different TMSPM molar ratios. (**c**) Transfer characteristics for ZnO-based transistors with PMMA–ZrO_2_ as gate dielectric hybrid films at different TMSPM molar concentrations. Reproduced with permission from [126], Copyright 2017, American Chemical Society.

Poly(methyl methacrylate) (PMMA) is an important thermoplastic polymer with excellent transparency, a refractive index of n = 1.49, good chemical resistance, thermal stability, mechanical flexibility, low cost, and a lower dielectric constant (2.9) than that of silicon oxide material (3.9) [123,124,125,126]. As a result, PMMA has been mixed with high-k inorganic nanoparticles such as ZrO_2_, Al_2_O_3,_ and TiO_2_ to provide high optical transparency, low weight, mechanical flexibility, and formability [14,127,128]. The low temperature deposition process towards PMMA-ZrO_2_ nanocomposites as dielectric gate layers has been reported [129]. Intriguingly, to prevent phase separation, inorganic oxides were cross-linked with PMMA and trimethoxy-silyl-propyl-methacrylate (TMSPM) molecules that are chemically compatible with both inorganic and organic phases. In greater detail, TFT devices with a ZnO/PMMA-ZrO_2_/ITO/glass structure (Figure 7a) were fabricated and their electrical properties, such as threshold voltage, channel mobility, and *I*_on_/*I*_off_ current ratio, were investigated. A hybrid dielectric layer was prepared via a sol−gel reaction among zirconium propoxide (ZP), TMSPM, and methylmethacrylate (MMA) precursors at variable TMSPM molar ratios. The devices fabricated with 0.3 M TMSPM exhibit a mobility of 0.48 cm^2^/V s, on/off ratio of 10^6^–10^7^, and a threshold voltage of 3.3 V. The leakage current density increases from 10^−6^ to 10^−5^ A/cm^2^ as the amount of TMSPM content increases in the hybrid insulating layer, as illustrated in the current density versus electric field characteristic curves (Figure 7b). Importantly, the threshold voltage of the devices decreases from 3.3 V to 0.9 V with an increase in the TMSPM amount from 0.3 M to 0.75 M, as measured by transfer curves in Figure 7c. This feature is advantageous for low power consumption.

**Figure 8:**
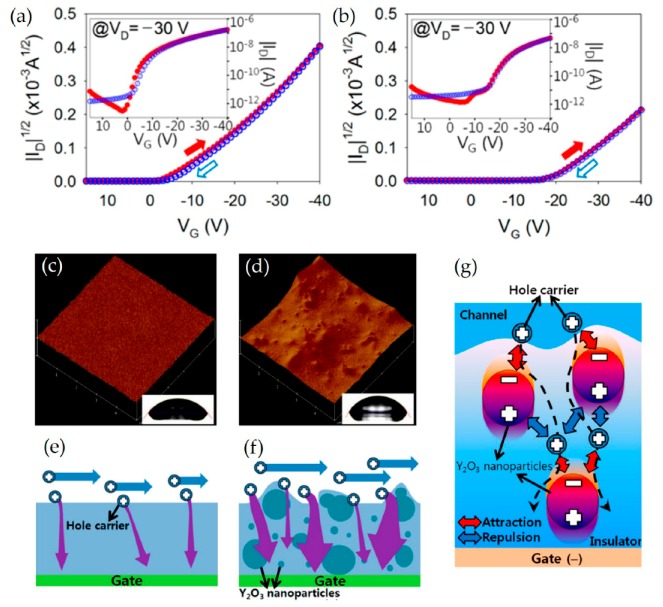
|I_D_|^1/2^ vs. V_G_ plots of TIPS-pentacene TFTs with the (**a**) c-PVP/Y_2_O_3_ composite and (**b**) c-PVP gate insulators. AFM images of the (**c**) c-PVP and (**d**) c-PVP/Y_2_O_3_composite films. The insets show the contact angles on both films. Leakage current paths through the (**e**) c-PVP/Y_2_O_3_ composite and (**f**) c-PVP gate insulators. (**g**) Possible interaction between the holes and the Y_2_O_3_ nanoparticles in the c-PVP/Y_2_O_3_ composite insulator. Reproduced with permission from [131], Copyright 2016, MDPI, Basel, Switzerland.

Yttrium oxide (Y_2_O_3_) nanoparticles exhibit a wide band gap of 6.0 eV, which is advantageous to the aspects of illumination stability of TFTs [130]. In this context, TFTs were fabricated on polyimide (PI) substrates using cross-linked poly(4-vinylphenol) (c-PVP)/ Y_2_O_3_ nanocomposites as gate insulators [131]. The architecture of the flexible devices was Ag/6,13-bis(triisopropylsilylehtynyl)pentacene(TIPS-pentacene)/ c-PVP:Y_2_O_3_/c-PVP/PI. In greater detail, the cross-linkable PVP was prepared by dissolving PVP and a cross-linking agent, (methylated poly(melamine-co-formaldehyde), MMF) in propylene glycol methyl ether acetate (PGMEA). TFTs with c-PVP:Y_2_O_3_ hybrid dielectric exhibited an on-state drain current of −0.165 μA at a gate voltage of −40 V, which is higher than that of devices with only c-PVP (−0.0462 μA), as depicted in Figure 8a,b. However, as illustrated in Figure 8c,d the c-PVP/Y_2_O_3_ composite films exhibited a higher roughness compared to the c-PVP films, leading to a larger interference in hole conduction at the interface between the insulator and the semiconductor. Additionally, c-PVP: Y_2_O_3_-based TFTs exhibited a greater number of leakage paths for the gate current compared to c-PVP-based TFTs, possibly owing to several interactions like i) the attraction of hole carriers by the highly polarized Y_2_O_3_ nanoparticles, ii) flow along the direction of the gate electric field, and iii) repulsion by the positive side and attraction by other adjacent side of the Y_2_O_3_ nanoparticles (Figure 8e–g).

Kim et al. have introduced TiO_2_-polymer composites via cross-linking reactions of these two constituents with low surface energy which allows vertical growth of organic molecules (e.g., pentacene) [132]. In greater detail, a TiO_2_ precursor (titanium(IV) butoxide and acetyl acetone) and poly(4-vinylphenol) (PVP) solution (PVP, poly(melamine-co-formaldehyde) methylated/butylated and propylene glycol methyl ether acetate (PGMEA) solvent) mixture were spin-coated on ITO substrates and then annealed at 200 °C for 1 h. Interestingly, poly(melamine-co-formaldehyde) methylated/butylated acts as the cross-linker, which reacts with the hydroxyl group of the PVP and the ligands of the TiO_2_, forming a dense structure. The resulting device exhibits a charge carrier mobility of 0.105 cm^2^ V^−1^ s^−1^, on/off ratio of 10^3^, and a leakage current of 10^−7^ A cm^−2^ at ±5 V due to such a dense structure. Furthermore, this homogeneous TiO_2_-polymer composite solution is stable in ambient conditions. Bang et al. fabricated bottom-gate ZnO-thin film transistors using PVP/Al_2_O_3_ dielectrics and investigated the effects of an organic/inorganic dielectric on device performance [133]. The leakage current of the PVP/Al_2_O_3_ dielectric improved by three times over the PVP counterparts. The saturation mobility of PVP/Al_2_O_3_ TFTs also improved from 0.05 to 0.8 cm^2^ V^−1^ s ^−1^ compared to PVP TFTs. 

**Figure 9:**
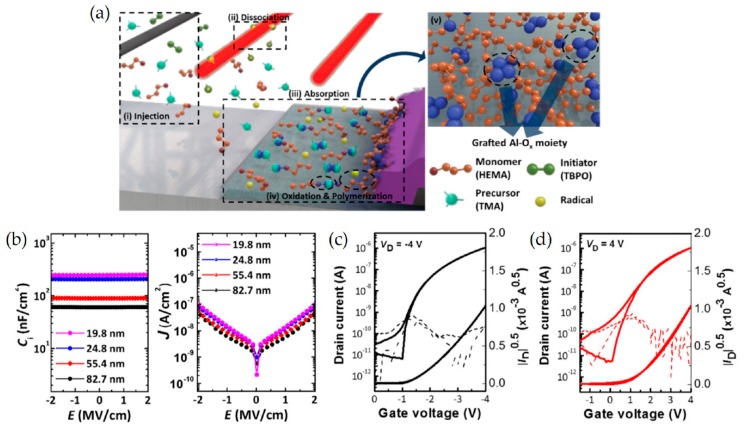
Vapor-phase synthesis of organic–inorganic hybrid dielectrics via iCVD. (**a**) A schematic of the synthesis process: (**i**) Vaporized monomers, organometallic precursor, and initiators are injected. (**ii**) The initiators were thermally decomposed near the heated filament to form radicals (red lines), which are positioned away from the substrate. (**iii**) Monomers, precursor, and radicals were absorbed on the heated substrate. (**iv**) The adsorbed monomers were polymerized and simultaneously reacted with inorganic precursors. (**v**) Uniform dispersion of the inorganic oxides can be achieved in the polymer matrix. (**b**) C_i_–E(left), and J–E(right) characteristics of the MIM devices with the hybrid dielectrics (Al concentration: 17.8%) with various thicknesses of 82.7, 55.4, 24.8, and 19.8 nm, respectively. Charge-transfer characteristics of the (**c**) pentacene and (**d**) PTCDI-C13 OTFTs, respectively. Hybrid films 25 and 34 nm thick were used as the gate dielectric for pentacene and PTCDI-C13 OTFTs, respectively. Reproduced with permission from [134], Copyright 2018, American Chemical Society.

Despite superior mechanical flexibility, organic materials as gate insulators, such as poly-4-vinylphenol (PVP) and polymethyl methacrylate (PMMA), exhibit very low capacitance compared to inorganic dielectrics. In this context, several approaches to improve the capacitance have been introduced. This includes reducing the thickness of dielectric films and incorporating high-k inorganic nanoparticles. However, the use of ultra-thin organic dielectrics often resulted in structural imperfections, producing current leakage. Kim et al. proposed a novel vapor-phase synthesis method to form an ultrathin, homogeneous, high-k organic−inorganic hybrid dielectric [134]. Hybrid dielectrics are synthesized via initiated chemical vapor deposition (iCVD) in a one-step manner (Figure 9a). This method utilizes 2-hydroxyethyl methacrylate and trimethylaluminum as the monomer and the inorganic precursor, respectively. A uniform and defect-free hybrid dielectric layer with precise thickness below 20 nm and composition can be produced. The hybrid films are formed via following subsequent steps—the injection of vaporized monomers, precursors, and initiators, the thermal decomposition of initiators to form free radicals, the adsorption of monomers and precursors, and free-radical polymerization of monomers. The hybrid dielectric exhibits a high k-value of 7 and a low leakage current density of less than 3 × 10^−7^ A/cm^2^ at 2 MV/cm, even with a thickness of less than 5 nm. The capacitance (*C*_i_) versus electric field and the current density (*J*) versus electric field characterizations corresponding to varying hybrid film thicknesses were also investigated, as illustrated in Figure 9b. As the thickness decreases, the *C*_i_ and *J* values reach 250 nF/cm^2^ and 1 × 10^−7^ A/cm^2^, respectively. The n- and p-type OTFTs were fabricated using the hybrid dielectric deposited via the iCVD process and their charge-transfer curves were studied, as depicted in Figure 9c,d. The hybrid dielectric offered a superior interface between the channel and dielectric and thus induced ideal charge-transfer characteristics. Both n- and p-type OFETs with the hybrid dielectric exhibited no apparent hysteresis and a low leakage current density (<3 × 10^−7^A/cm^2^ at 2 MV/cm). Furthermore, the dielectric layer exhibited improved chemical stability without any degradation in its dielectric performance. Interestingly, the hybrid dielectric layer retained its excellent dielectric performance under tensile strains of up to 2.6%.

The effects of the device architecture on indium zinc oxide (IZO) TFTs with poly(4-vinylphenol-co-methylmethacrylate) (PVP-co-PMMA) gate insulators were investigated [135]. The top gate IZO TFTs exhibited the improved μ_FE_, SS, *V*_th_, and good Ion/off ratio of 8.5 cm^2^ V^−1^ s^−1^, 2.0 V per decade, -10.0 V, and 10^7^, respectively, compared to the bottom gate IZO TFTs (μ_FE_, SS, V_th_, and Ion/off ratio were 9.0 cm^2^ V^−1^ s^−1^, 5.0 V per decade, −12.5 V, and 2 × 10^5^, respectively). This is attributed to the energetic ion bombardment in the polymer gate dielectric layer during the sputtering process. The device performance can be further improved by doping the hybrid PVP-co-PMMA gate dielectric with ZrO_2_: the μ_FE_, SS, V_th_ and I_on/off_ ratio in this case were 28.4 cm^2^ V^−1^ s^−1^, 0.70 V per decade, −2.0, and 4.0 × 10^7^, respectively. 

**Figure 10:**
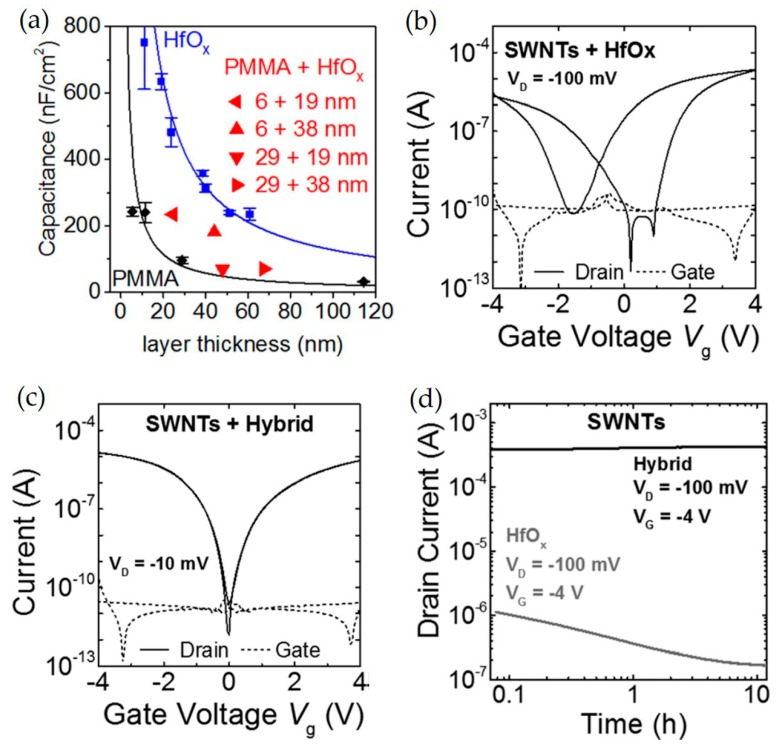
(**a**) Capacitance as a function of total layer thickness for hafnium oxide, PMMA, and hybrid dielectric. Transfer characteristics of network SWNT FETs with (**b**) HfO*x* and (**c**) hybrid dielectric. Channel width/length ratio and channel lengths were 125 and 40 μm, respectively. (**d**) Bias stress tests of SWNT-based transistors with different dielectrics. Reproduced with permission from [136], Copyright 2015 American Institute of Physics.

To improve surface contact with organic molecules and increase dielectric properties, a bilayer structure was introduced. For example, Held et al. fabricated a bilayer hybrid dielectric consisting of a high-k hafnium oxide (HfO*_x_*)/thin PMMA layer with a donor-acceptor polymer, poly(2,5-bis(2-octyldodecyl)–3-(5–(thieno[3,2-b]thiophen-2,5-yl)thiophen-2-yl)–6-(thiophen-2,5-yl)pyrrolo[3,4-c]pyrrole-1,4(2H,5H)-dione) (DPPT-TT) or single-walled carbon nanotubes (SWNTs) as the semiconductor [136]. PMMA layers were spin-casted and hafnium oxide layers were deposited via ALD. The resulting FETs exhibited drastically reduced operating voltages. The PMMA/HfOx hybrid dielectric exhibited low-voltage operation, well-balanced charge carrier transport, low trap densities, and excellent bias stress stability as PMMA ensures a low density of trap states at the semiconductor dielectric interface and HfO*_x_* layers provide high capacitance (Figure 10a). Moreover, the effects of a hybrid dielectric layer for SWNT-FETs were investigated. The SWNT-FETs with only HfO*_x_* dielectric layer exhibit strong threshold shift and hysteresis, as observed in the transfer characteristics (Figure 10b). In contrast, ambipolar transfer characteristics without hysteresis was observed in SWNT-FETs with the hybrid dielectric (Figure 10c). According to bias stress tests, SWNT-FETs with hybrid dielectric exhibit constant on-currents without any noticeable degradation over 10 h, while SWNT/HfO*_x_* FETs suffer an on-current decay of an order of magnitude recorded in Figure 10d.

High performance low-voltage pentacene-based organic TFTs with pentacene/PMMA/Al_2_O_3_/ITO architecture were fabricated and their electronic characteristics were investigated [137]. In this study, a high-k metal oxide dielectric, Al_2_O_3_, was used due to its excellent dielectric constant (k = 7.0~9.0) and large bandgap (*E*g = 8.45~9.9 eV). PMMA renders improved interfacial properties between Al_2_O_3_ and organic pentacene. The OFETs with only an Al_2_O_3_ layer exhibited a field-effect mobility of 0.65 cm^2^/Vs, a threshold voltage of −0.6 V,*I*_on_/*I*_off_ ratio of 4 × 10^3^, and a sub-threshold swing of 0.45 V/dec, at operating voltages as low as −4 V. After being modified by PMMA, the mobility increased from 0.65 to 0.84 cm^2^/Vs. 

**Figure 11:**
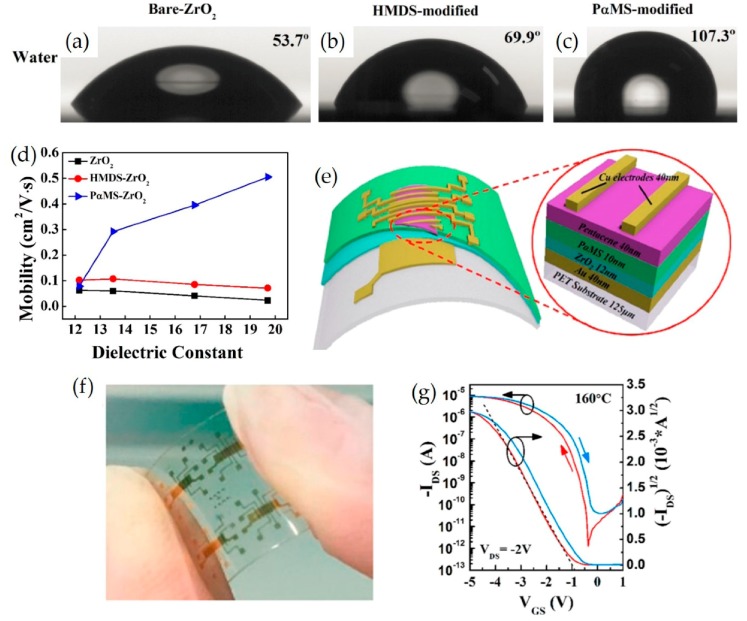
Water contact angles of (**a**) bare-ZrO_2_ surface, (**b**) HMDS modified surface, (**c**) PαMS modified surface. (**d**) Field effect hole mobility as a function of ZrO_2_ dielectric constant for OFETs with different surface modifications. The mobility was calculated with V_G_ = −5 V and capacity density under *f* = 1 kHz. (**e**) Schematic diagram of the flexible OTFT fabricated on PET substrate and (**f**) the digital photograph of the flexible OTFTs. (**g**) I_DS_–V_GS_ transfer curves of a ZrO_2_-OFET constructed on PET flexible substrate. The channel width and length of the transistor are 750 μm and 50 μm, respectively. Reproduced with permission from [138], Copyright 2016 American Chemical Society.

Poly(α-methylstyrene) (PαMS) was also applied on top of zirconium oxide (ZrO_2_) layers to improve the quality of the interfaces between ZrO_2_ and organic semiconductors [138]. In greater detail, a ZrO_2_ film was synthesized on Si via a chemical solution process and annealed at temperatures between 400 and 700 °C. PαMS or HMDS layers were then spin-casted and made to undergo vacuum evaporation with pentacene. It was found that the surface modifications greatly affect the electrical performance of the ZrO_2_ OTFTs. The surface energy deceased after surface modification and the calculated values are 43.9, 37.8, and 35.5 mJ/m^2^ for bare-ZrO_2_, HMDS-ZrO_2_, and PαMS-ZrO_2_, respectively, as depicted in Figure 11a–c. The PαMS modified devices exhibited a higher carrier mobility and on/off ratio than those fabricated with bare ZrO_2_ and HMDS-coated ZrO_2_ because the PαMS/ZrO_2_ layers provide a low surface energy and thus promote the growth of large pentacene crystals. In particular, the carrier mobility of the devices with PαMS-modified ZrO_2_ were observed to increase remarkably from 0.08 to 0.51 cm^2^/Vs, whereas the carrier mobilities of the devices with bare ZrO_2_ and HMDS-modified ZrO_2_ remained at values of ~0.06 and ~0.11 cm^2^/Vs, respectively, while the dielectric constant of ZrO_2_ was increased from 12.17 to 19.70 (Figure 11d). Furthermore, PαMS/ZrO_2_ OTFTs fabricated on flexible polyethyleneterephthalate (PET) substrate were demonstrated, as depicted in Figure 11e,f. The flexible OTFTs exhibited typical I_DS_-V_GS_ curves of the ZrO_2_-OFET, exhibiting a ~10^5^ on/off-current ratio between +1 V and −5 V of V_GS_ (Figure 11g).

Ha et al. have reported on low-voltage OTFTs employing solution-processed hybrid bilayer gate dielectric of high-k ZrO_2_ and low-k amorphous fluoropolymer, CYTOP [139]. The thin hydrophobic CYTOP layer repels aqueous molecules from an organic active layer. Therefore, such device architecture improves electronic characteristics including field effect mobility (from 0.18 to 0.28 cm^2^/Vs), threshold voltage (*V*_th_, from 0.4 to -0.1 V), and sub-threshold (S.S., 0.57 to 0.28 V/decade) compared to only high-k ZrO_2_ devices. The reduction in defect-states at the interface suppresses photo-induced hysteresis and enhances the stability of device performance against electric bias-stress. 

## 4. Summary and Outlook

In summary, we first give an overview of the development in polymer/metal oxide nanocomposites for applications in flexible charge transport channels and dielectrics. Recently, metal oxides (MOs) have been fabricated via vacuum-based techniques including pulsed laser deposition (PLD), atomic layer deposition (ALD), magnetron sputtering, and e-beam evaporation, for use in flexible and transparent charge transport channels. Despite their ultra-thin width, only inorganic MO films are vulnerable to repeated mechanical deformation. As a response to low mechanical durability and flexibility, hybrid polymer/MO nanocomposites have been introduced. Hybridization with soft organic materials have proven to be an effective strategy that not only offers mechanical flexibility but also enables solution-based fabrication. 

Organic dielectrics have garnered substantial attention owing to their flexibility, mechanical stability, solution processability, and excellent compatibility with flexible organic substrates. However, the low k values of such materials prohibit their application in practical electronic devices. Thereby, high-k inorganic MOs have been employed as fillers. Considering that most of the flexible substrates and semiconductors are organic materials, hybrid gate dielectrics tend to provide good compatibility with organic substrates and semiconductors. 

Despite significant advances in flexible electronics by using polymers, many challenges remain to be surmounted, including poor mechanical durability, low charge-carrier mobility, and low dielectric constants. However, we believe that hybrid nanocomposites will reach their full potential in flexible electronics in the near future, as various methods to overcome their weaknesses are being continuously explored.
